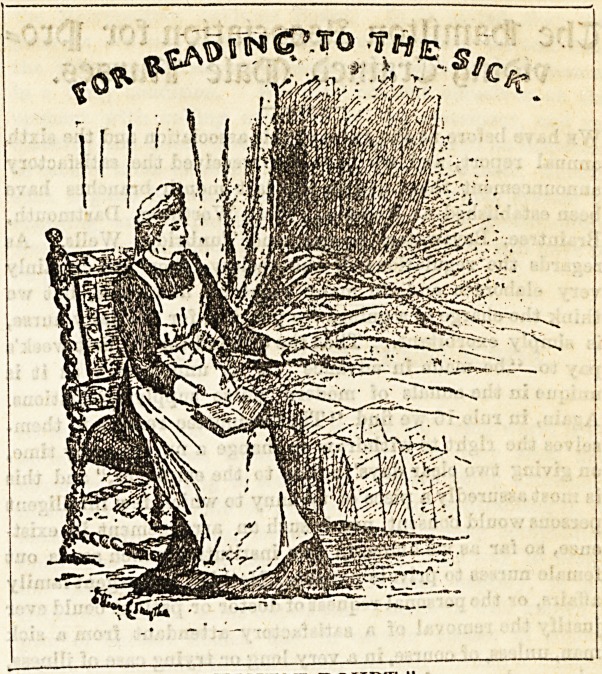# The Hospital Nursing Supplement

**Published:** 1892-07-02

**Authors:** 


### "The Hospital" Nursing Mirror.

Being the Extra Nursing Supplement of "The Hospital" Newspaper.

Contributions for this Supplement should be addressed to the Editor, The Hospital, 140, Strand, London, W.C., and should have the word "Nursing" plainly written in left-hand top corner of the envelope.

### En Passant.

**Royal South Hants Infirmary, Southampton.**—The three small houses in Fanshawe Street, which now constitute the nursing home, are a success in every way, and fully justify the money laid out on them. The training of probationers at this infirmary is as yet in its infancy, but so far as it has gone, the system has answered well. Mr. H. J. Buchan, whose death left a blank at this hospital not easily filled, lately gave £50 to the Nurses' Pension Fund.

**The Registration of Midwives.**—It has been reported by the Select Committee appointed to consider this Bill that the evidence taken shows that there is at present serious and unnecessary loss of life and health and permanent injury to both mother and child in the treatment of childbirth, and that some legislative provision for improvement and regulation is desirable. They have also had evidence showing that there is a wide field for training in midwifery now unused connected with parish infirmaries and home practice in populous places, and they recommend a continuation of the inquiry by the future Parliament.

**He or She?**—A request was received the other day, at the Hamilton Association, for a nurse to be sent to take charge of an elderly gentleman who was very ill. Of course, some one went immediately to the address given, and when he arrived, great dismay was evinced by the patient's friends at finding they had unwittingly summoned a man instead of the woman with whom they had hitherto exclusively associated the honoured title of nurse. Happily, the attendant proved a complete success, and a most satisfactory commendation of his general conduct and efficiency was eventually received by the Secretary. There are many pleasant anecdotes in circulation as to the surprise expressed in various households regarding the adaptability and general "handiness" of some men-nurses.

**Private Nurses in Dublin.**—We hear that it is proposed to establish in Dublin a Home for Private Nurses, working on their own account, and receiving their own fees. Such a home ought, it is believed by those starting the scheme, to be a great help to both private nurses and medical men, who are often inconvenienced by the delay necessarily incurred where nurses are scattered about in different lodgings. In addition to the home, a registry of nurses will be kept for those who, from various circumstances, may not wish to reside in the home, and a club room for nurses attached to hospitals and nursing institutions is also part of the plan, and a meeting of Lady Superintendents of hospitals and nursing institutions was held on Wednesday last at the Home Hospital, 74, St. Leeson Street, to discuss the scheme. Miss Huxley (Sir Patrick's Dunn's), Miss Poole (The Adelaide), Mrs. Burton (Hollis Street Nursing Institution), Miss Wall (St. Mark's), Miss Singleton (Home Hospital), and others were present. Mrs. Calcutt, who will manage and who is starting the Home, read a paper explaining her scheme, which was approved of by all present. We should think the Dublin nurses might get some good hints from the Nurses' Residential Club, in Charlotte Street, as to the working of such a home, and if the whole is under trained supervision, and only fully trained nurses are taken on the register, it ought to be a success.

**St. Kilda.**—The first visit this season to the island was made last week. The population now numbers 71. A change is to be made in the nurse at St. Kilda, and we are sorry to hear Mrs. Chisnhall is leaving; two epidemics during the winter, one of typhus and one of whooping cough, gave her plenty of work. Mrs. Chisnhall has been instructing the girls in sewing, an almost neglected art in the island, and has done much good work during her two years. She was sent out by the Glasgow Sick Poor and Private Nursing Association.

**Stockton and Thornaby District Nurses.**—A special meeting of the Council of this association was held last week to open the new Home for the District Nurses. Lady Londonderry presided, and in a short speech congratulated the subscribers on the home, and spoke with warm approval of the decision that the working men, who are such good subscribers, are to add to their representation on the Council by five members. In future the nurses are also to be allowed to go to those who cannot afford the prices of ordinary private nurses, but who will be glad to pay a small fixed charge for the district nurses' services. The home is open for a week or so for the inspection of subscribers.

**Short Items.**—The Nurses' Institution, at Torquay, has been in want of funds, and the 30,000 inhabitants are to be canvassed for the sum of 2d. each.—The special service at St. Paul's Cathedral, in aid of the East London Nursing Society, was well attended, and the Rev. Prebendary Jones preached the sermon.—The Cathedral Nurse and Loan Society, at Newcastle, has a record of a year's good work; the Convalescent Home at Shotley Bridge has received 98 patients, and another home is to be opened at Hexham.—The Forfar district nurse will begin work in September.—The Nurses' Residential Club in Charlotte Street is a great success, and the demand for lodgings exceeds the supply.—Miss Sutliffe, of the New York Hospital, and her sister, Miss Ida Sutliffe, are in England for a holiday.—Some of our nurse friends are going to Jersey, and we will gladly supply any information about expenses, &c.

**Allhallows, Ditchingham.**—This little hospital, though not much known beyond its own locality, has been doing good work for the last nineteen years, under the superintendence of the Allhallows' Sisterhood, by whom it has been brought into an almost perfect state of efficiency. Situated in the healthiest part of Norfolk and on the borders of Suffolk, it gets the pure air from the North Sea, which renders it a fit place for a convalescent home. There are now seven incurable inmates; two very old women and one young girl having died lately, the last of consumption, for which she had been admitted; but there is a floating population of sick folk who come thankfully from the adjacent villages to receive the comforts and attention to be found at this little oasis. Three ladies, paying patients, were received last year, two of them came to undergo serious operations, which were successfully performed, and all three left quite restored. The Hospital is supported by voluntary contribution. At present the great want is a long invalid couch on which the incurable patients might be wheeled about the grounds and get a little change of scene, several of them having been in their beds for over fifteen years. Such a present, or subscriptions towards one, would be thankfully received by Sister Lucy, Allhallows, Ditchingham, Norfolk.

### Ventilation, Disinfection, and Diet.

By P. Caldwell Smith, M.D.

### XII.—DIET AND DIETARIES.

Classification of Foods—Nitrogenous Foods—Different Kinds of Albumen—Peptones—Use of Albuminous Foods—Carbohydrates—Fats—Mineral Matters—Water—Accessory Foods—Quantity of Proximate Principles Required.

The animal economy has been compared to a steam-engine, in which for a certain amount of work done, or power exerted, a certain amount of coal has to be burnt and a certain amount of water evaporated. The human body, however, is a very much more complicated piece of machinery than any steam engine, while the fuel which is required is much more elaborate. The food which we eat is analogous to the coal and water of the steam-engine, and it is necessary for our purpose that we enquire (1) what are the main scientific divisions of foods? (2) what are their uses? and (3) the construction of dietaries on these?

Foods may be broadly classified as follows: (1) Nitrogenous, meaty, or albuminous food; (2) non-nitrogenous, which may be divided into *(a)* starches or carbohydrates, and *(b)* fats, all including oils, animal and vegetable fats; (3) mineral matters; (4) water; (5) accessory foods as tea, coffee, cocoa, alcohol. We shall take up these in detail. (1) Nitrogenous foods; these are also called albuminous principles or proteids. They are called albuminous because the albumen, or white of the egg, is taken as the type. There are a number of different forms of albumen, which differ slightly in their chemical relations, but for our purpose it will be sufficient to mention merely the different kinds of albumen. (1) Egg albumen; (2) serum albumen, got from blood of man and animals, and also found in the urine of patients suffering from Bright's disease of the kidney; (3) albumen found in ordinary flesh, and (4) casein or albumen found in milk.

The above are the principal albumens present in animals. There are also some found in the vegetable kingdom as gluten, which occurs in all cereals, as corn and wheat, and legumen, found in beans, peas, lentils. Then we have what are called gelatinoids, as chondrin and gelatin, found in bone and gristle; but these are not important as foods, as they cannot be utilised as nourishment.

Now these albumens when taken into the stomach cannot be digested and assimilated before they are acted on by the juices of this organ and of the pancreas. They are by these converted into what are called *peptones*, which are absorbed. What is the use of these albuminous foods? Could we do without them? Could life be sustained if we were deprived of them? are very important questions. These may be answered thus, that this form of food is absolutely necessary for the growth and repair of the system. Nitrogen, which is one of the main constituents of albuminous or nitrogenous foods, is present in the brain, the nerves, the blood, and some must therefore be supplied to make up for the waste which goes on constantly in the system. The greater amount of nitrogen we take we get from animal food, as beef, mutton, &c.; although, of course, eggs and milk contain a certain proportion. If too little of this form of nourishment is supplied, loss of weight, bloodlessness, want of energy and life result; on the other hand, if too much be given, that is, if too much animal food be eaten, we are liable to produce general fulness of the system, gout, and disorders of liver, not to speak of indigestion, with its numerous symptoms and results.

In the next division we shall first speak of what is called the carbohydrates, or starches. These include the starches proper as found in potatoes, rice, sago, tapioca, arrowroot; the sugars, including cane sugar, honey sugar, grape sugar, beet sugar, and sugar of milk, and the gums or mucilages of fruits and vegetables. These are required in the system, as they produce energy, and what is most important, animal heat. Some portion is utilised at once in the system, while another portion is stored up in the liver, from which it is withdrawn when required. We see then that the liver is not only necessary for the secretion of bile, but also as a kind of storehouse for the sugar.

The fats are necessary for the formation of the fat in the body, although this may also be formed from the starches. The fat is absorbed in great part as such by the intestines, while some of it is formed into a kind of soap by the action of the pancreatic juice, and then absorbed.

(3) Mineral matters, as common salt, phosphates of lime, &c., are also necessary for the support of the economy.

(4) Water.—The amount of water taken by an individual in health will, of course, vary much according to habit, time of year, and amount of exercise taken, a good average being from 40 to 50 ounces, while a certain amount of this substance is contained also in some of the foods we eat. The total amount of water then taken into the system in 24 hours varies from 70 to 80 ounces. Our system can stand deprivation of food much longer than total deprivation of water. Some of the fasting men, as Succi, have refrained from food for fifty days, drinking water, however, pretty copiously. No one can stand the withdrawal of water for a day or two without suffering intense agonies. The stories we frequently read of shipwrecked crews proves this only too satisfactorily.

(5) Accessory Foods.—In this we may include alcohol, tea, coffee, chocolate, spices, &c. The precise mode of action of these substances is as yet not well known. They are not, however, to be regarded as being absolutely necessary for a complete dietary.

(6) Digestibility of Foods.—It is only by making very exact and elaborate experiments that this can be at all decided. With everything we eat there is always a certain amount thrown off as waste, and this varies very largely.

It seems tolerably certain that life cannot be supported for any length of time on any diet which does not contain albuminates, fats, carbohydrates, and salts, or what may be called the proximate principles of diet. For example, take the case of fat; it has been proved that animals may live without it for a certain time, but there is a great difficulty in excluding all the fat, as carbohydrates or starches can be converted into fats in the system.

We now pass on to consider the quantity of each of these proximate principles required in a diet. This is rather difficult, as different authorities give different amounts, and it is easy to see wherein the difficulty lies. No individual man, woman, or child requires the same amount. The proportion of each of these principles differs in childhood in comparison with adult life. A woman also requires less than a man. The calculation can only be given for average men, taking into consideration, in forming a diet for an individual, the physique and the nature of the work of each individual. The dietaries of women should be about $\frac{1}{10}$ less than those of men. A child at ten years will require about half as much food as an adult woman, and at about fourteen years nearly as much.

It may be given as a fair average that a man who is practically at rest requires nitrogenous food to the amount of 2·5 ozs., while at hard work it would have to be more than doubled, viz., 6 ozs.; that the fats require to be increased from 1 oz. at rest to $4\frac{1}{2}$ ozs. at hard work, while the carbohydrates are only increased from 12 ozs. at rest to 18 ozs. at hard work.

This shows that the amount of animal food must be largely increased at hard work, and this is in accordance with every one's experience. In hot climates again it would not be necessary to put nearly so much fat or nitrogenous matter, while the carbohydrate could be largely increased. In very cold weather a larger amount of fat may be taken.

### The Lords' Report on the Proposed Registration of Nurses and British Nurses' Association.

THE report of the Lords' Committee on the Metropolitan Hospitals ably sums up the question of the registration of nurses as proposed by the Royal British Nurses' Association, which they find will be no protection to the public, though it would tend to reduce all nurses to one common level.

PROPOSED REGISTRATION OF NURSES, AND BRITISH NURSES' ASSOCIATION.

An important question affecting the general position of nurses was brought forward in connection with the scheme proposed by the British Nurses' Association, for establishing a general register of nurses. A very broad division of opinion exists regarding the merits of that Association. Its objects, as stated by its advocates, are, "first, to unite trained nurses together in a purely professional union; secondly, to provide for the local registration of nurses under the control of medical men; thirdly, to help nurses in times of need or adversity; and, fourthly, to improve the knowledge and usefulness of nurses throughout the empire;" and its scheme is declared to be put forth "in conformity with a great public want and a widespread professional demand." This statement is traversed in a memorial which was signed by many members of the medical and nursing staffs, and of the governing bodies of hospitals and institutions for the sick in London and the provinces, and which was claimed to represent the majority of those who know most about nursing in this country. The memorial declares that the proposal if carried out "would lower the position of the best trained nurses, be detrimental to the advancement of the teaching of nursing, be disadvantageous to the public, and be injurious to the medical practitioner." A petition against the scheme, also largely signed, was presented to the Board of Trade.

OBJECTS OF BRITISH NURSES' ASSOCIATION.

The view taken by the promoters of the Association appears to be that the time has come when nursing should be constituted and legally recognised as a distinct profession, with a central controlling body of its own; in short, that the nursing profession should be governed on much the same lines as the medical profession. The nurses' register would resemble the medical register, and the General Nursing Council would take cognisance of the conduct of all nurses, and would have the same power to strike their names off the register for misconduct, as in the case of the medical profession is exercisable by the General Medical Council. The ultimate object appears to be (whether or not this could be carried into effect at once) to obtain statutory power to prevent any public or private institution sending out women to nurse the sick, who were not registered by a registration board, composed of medical men and hospital Matrons, or at all events to prevent unregistered women calling themselves trained nurses. But whether or not there were any such express prohibition, it was thought that a Registration Board constituted under Royal Charter or Act of Parliament would have such prestige that the public would decline to employ unregistered nurses. It was claimed that some of the hospitals and many medical officers of hospitals were in favour of registration. The immediate advantage which the public would gain from it was said to be that a reference to the register would at once show whether a woman was a trained nurse or not, and whether she was known to have ever done anything rendering her unworthy of employment, because the name of a nurse would, on sufficient cause shown, be removed from the register; the witness further said "it was a very common fraud to steal or forge a hospital certificate." No hospital is responsible for a nurse once she has left the hospital service; but a General Nursing Council or Registration Board would be responsible to the general body of nurses, and to the public; to prevent any woman who proved herself unworthy of trust going on with the work, they would take her name off the register.

OBJECTIONS TO BRITISH NURSES' ASSOCIATION.

The main point alleged against the British Nurses' Association by its opponents is that it places good and bad nurses on a level. It is urged that neither the completion of a certain period of training nor the passing of a theoretical examination is sufficient guide to the practical fitness of a woman for a nurse's work. Only the institution which has actually trained the nurse, and in which her qualities are recorded after long personal observation, can be in a position to give such a guarantee of her capacity as will be of any practical value. If, for example, a member of the public goes to such a general register for a nurse, he gets someone who has passed through a certain curriculum; if he applies to any nurse-training hospital, he gets a nurse selected for the particular case, and backed by the authority and reputation of the hospital which sends her out. It was further said (in the interests of the medical profession) that the grant of a sort of diploma to nurses might lead many people to seek a nurse in case of illness and not a doctor; such a result, it was thought, would be injurious also to the interests of the nurses themselves.

THE PUBLIC ARE ALREADY PROTECTED.

Under the existing system it is argued that the public have adequate protection in their power to call for a nurse's certificate before employing her, and to obtain particulars from the hospital which gave it her; that this security would under the registration scheme be lost, and that women, whom no hospital would recommend, would get themselves registered and appear to the public on the same level as the best nurses. It was suggested that an official list (if it were needed) could be compiled giving the names of all nurses on the books of the several training hospitals.

REGISTRATION NO PROTECTION TO THE PUBLIC.

A point very strongly urged is that the character of the woman herself is a most essential matter in regard to a nurse; much more so in the case of a nurse than of a doctor. The Association professes to require evidence of character (by the production of recent testimonials) before it will put a nurse on its register, and to register only women who have had three years' hospital training, but it appears that women are registered who have not completed their full period of training at any one hospital, and of whom it is not known whether they have proved themselves competent or otherwise. The Association complains that a hospital certificate, once given, cannot be withdrawn, whereas a name will be removed from the register whenever a nurse is proved to have forfeited her good character, legal proof being admittedly exceedingly difficult. But it is evident that this course cannot be taken except on clear proof of actual crime or misconduct, and therefore it is no protection to the public from mere incompetency. It was admitted that a woman might go through three years' training at a hospital, and get her certificate, and yet be a very indifferent nurse, and be known at the hospital to be so; but the public who read her name in the register, would suppose her to be competent unless the register clearly stated that it did not guarantee the efficiency of its nurses. On the other hand, if the Association disclaims responsibility for the efficiency of the nurses whom it registers, it seems difficult to understand wherein lies the security which it offers to the public.

MISS NIGHTINGALE'S OPINION.

Mr. Rathbone, speaking on behalf of the Nightingale Training School, in opposition to the Association, quoted from a letter written by Miss Nightingale on this subject: "You cannot select the good from the inferior nurses by any test or system of examination. But, most of all, and first of all, must their moral qualifications be made to stand pre-eminent in estimation. All this can only be by secured by the current supervision, tests, or examinations, which they receive in their training school or hospital, not by any examination from 'a foreign body' like that proposed by the British Nurses' Association. Indeed, those who came off best in such would probably be the ready and forward, not the best nurses."

### Colonies for Epileptics.

It must be at least seven years since the subject of Colonies for Epileptics was first discussed in England, and though the scheme has flourished in Germany and America, and has been universally praised here, it has not yet been put to practical use. But there seems some hope that before another seven years are over something will be done, for a fairly strong Committee have taken the matter in hand, and have issued an appeal for £10,000 with which to start it. On this Committee are Dr. and Mrs. Ferrier, and probably no name carries greater weight amongst those wise in nervous diseases than Dr. Ferrier; Mr. C. S. Loch is also on the Committee, and Dr. Buzzard and Miss Nina Paget. The Honorary Secretary is Miss Burdon-Sanderson, Branksome, Greenhill, Hampstead, N.W., to whom contributions may be sent, and who will supply information to those interested in the plan.

All those who have worked much among the poor must have found that the hardest cases to relieve are those of epileptics. Full of energy and keen intellect between whiles—these poor wretches are never sure of themselves—they are cursed with the perpetual fear that at any moment they may fall in a fit, and continuous employment is almost impossible to them. What man will retain as clerk one who is subject to fits? What woman will retain as servant one who may any day fall into the fire whilst cooking, or fall in the street while carrying the baby?

The humiliation of spirit caused by this illness is not the least bitter part of it; the natural desire is to hide away, to let no stranger intermeddle with this grief. And yet the epileptic is ever at the mercy of strangers, and tender though that mercy often is, it cannot do away with the feeling of disgrace and helplessness which grows with the return to consciousness. No wonder the ancients said that epileptics were "tormented of the devil." Verily, no greater torment could be devised. There exists a wide belief that epileptics are also generally idiots; this is far from being the case, for we could tell of a man who suffers from epilepsy whose brilliant intellect is unsurpassed.

When an epileptic is also an idiot, the case is one which can be received in an asylum, and is not difficult to deal with; it is for the epileptics who, when in health, have full possession of their faculties, that practically no provision exists in England. They can work, they can think, they can suffer; but work there is none for them, and, as a rule, they live hidden away in corners, kept out of sight by relatives who feel them a burden and a disgrace, and there they eat out their hearts in misery and helplessness. In Germany exists a colony of 1,100 persons, all subject to epileptic fits, and yet busily and happily employed in farming, market gardening, bookbinding, washing, &c., all under careful supervision, and according to a well thought out scheme. The colony is not quite self-supporting, but all are busy; none are allowed to feel themselves useless or cumberers of the ground. It has also been found that thus grouping these sad cases together, instead of being harmful, induces feelings of contentment and hopefulness. It is on the lines of this settlement that the industrial colony in England is to be started if only the public will come forward and help.

The English are a proud race, and keep their family skeletons well hid, as a rule. In how many homes does this skeleton take the terrible form of some poor epileptic or idiot, kept always at the country house, or perhaps boarded with a doctor and visited only once or twice a year? To those suffering from such a skeleton we would suggest to give help for the proposed colony, for it will be a blessing, not only to the very poor and destitute, but paying patients will also be taken, suitable work will be provided for them, and life will offer them once more work, companionship, and hope.

Nervous diseases are terribly on the increase owing to our present manner of life. Crowding into towns, over brain work, &c. Nature shows us the cure—a return to country life and manual labour, a return which is practically the only hope for those stricken with epilepsy. Are we to refuse them this chance of cure?

### The Toronto General Hospital Training School.

This school has entered on its eleventh year of existence, having started in 1881 with sixteen probationers, who were already members of the staff of the hospital, but who undertook to stay two years as pupils of the school. Of this number only five completed their training, when they were examined orally and received the certificate and the badge, an engraving of which we give, which entitled them to call themselves trained nurses. In January, 1885, we find the staff consisting of a Superintendent, a supervisor of night nurses, seven certificated nurses in charge of wards, and twenty-seven pupil nurses. The nurses had to serve all meals in the wards and wash up the dishes, and for recreation they had an hour off duty each day, one afternoon each week, and half of each Sunday. There was a dining-room in the basement of the hospital, and bed-rooms wherever room could be found for them. Contentment reigned, and the nurses made the best of things, knowing that improvements were gradually increasing. In 1887 the Nurses' Home was finished, and the nurses nowadays are able to revel in a good dining-room and two sitting-rooms, where kind friends have placed a beautiful piano and a general and medical library. At the present time there are fifty-five pupil nurses in training, five probationers, and two permanent nurses. Examinations are held every six months, and the course of training comprises elementary anatomy, physiology, and hygiene, together with practical nursing, while lectures on various subjects are given which are calculated to make nurses more efficient. The practical training is the same as in our own hospitals, and the whole system enables nurses to discover the "wide difference both in kind and in degree between the knowledge necessary for a doctor and that necessary for a nurse." They learn, if they learn truly, that simple obedience must ever be their watchword.

This year a new pavilion in the hospital, containing nearly forty beds, has been set apart for gynæcological cases, which will be of great value to the school, and regular instruction in practical dietetics will be added to the course of instruction.

During the probationary month the applicant goes through an examination of the three R's and English dictation, and the creditable performance of the same is indispensable for a pupil. At the end of that period the probationer who is accepted enters the home and serves as a nurse; she receives three dollars a month the first year, and six dollars a month the second year, and those who complete the full course and pass the final examination are entitled to receive 25 dollars with their certificate. This remuneration is considered equivalent to the education and instruction received. The rules for the nurses are in the main very much the same as those of our well organised training schools.

The hours of duty for day nurses are from seven a.m. until seven p.m., and the yearly holiday is for two weeks. Up to the present time 131 women hold the certificate of the school, twenty-nine of these hold positions in hospitals, fourteen are married, five are foreign missionaries, and many are private nurses in various parts of Canada and the States. As is well known, the Pension Fund Scheme is securing many advocates across the ocean, and Dr. O'Reilly, Medical Superintendent of this hospital, is acting as honorary representative of the R. N. P. Fund for nurses in the Dominion of Canada.

The following are graduates of the school:

1883: Agnes Rose, Mary Graham, Elinor Potter, Margaret Maxwell, and Margaret Campbell. 1884: Annie Barton, Sarah Burrill, Mary Clark, Ann Denovan, Rosetta Pearson, Mary A. Orr, Henrietta Moote, Hannah Cody, Emily Brady, Effie Hewitt, Eliza Kennedy, and Jessie Duncan. 1885: Grace Dalgleish, Annie Hurst, Sarah Barge, Annie Boyd, Margaret Brown, Sarah Simpson, Elizabeth Jones, Kate Rogers, Sarah Clark, Sarah Johnston, and Catherine Greig. 1886: Alice Amos, Laura Whittaker, Margaret McMillan, Mary Kennedy, Barbara Allan, Mary Tipping, Mary Lowe, Jessie McLaren, and Gertrude Thorne. 1887: Keziah Underhill, Mary Yerex, Isabella Harsburgh, Sarah Gamble, Adelaide Sewell, Esther Kinsey, Grace Mowat, Minnie Barker, Kate Good, Theresa Miller, Christina Hall, Isabel Wamsley, Margaret Middlemas, Eliza Livsey, and Lizzie Gibson. 1888: Marion Wilson, Annie Coleman, Louise Eastwood, Hattie Sutherland, Ethel Woffingdin, Florence Bligh, Annie Carveth, Helen McKellar, Jessie Howard, Christina Mitchell, Margaret McDonald, Annie Littlehales, Louisa Phymister, Lizzie Howard, Annie Robinson, Margaret Gifford, Nellie Stowe, Christina McCormack, and Hannah Hollingworth. 1889: Elizabeth McKenzie, Bessie Sutherland, Margaret McIntosh, Carrie Watson, Jennie Graham, Nettie Lawder, Sarah Snyder, Agnes Boyd, Kate Anderson, Mary Steers, Helen Cameron, Fanny Tribe, Eliza Gordon, Carrie Smith, Agnes Pettigrew, and Florence Webster. 1890: Gertrude Osborne, Ada Marsh, Ida Moore, Nettie Haigh, Emma Rogers, Margaret Gourley, Margaret McKerricher, Nettie Ferguson, Kate McTavish, Augusta Blakeley, Elizabeth Senior, Annie Hollingworth, Janet Ardagh, Maggie Frazer, Anna Bartle, Carrie Bowman, Carrie Currie, Marguerette Clendenning, Annie Sutherland, Christina McKay, Gertrude Gallon, Annie L. Haigh, and Margaret Watson. 1891: Lilla Sheppard, Alice Lawson, Rachael Hanna, Kate Johnston, Agnes Kay, Leila Batty, Bessie Dewar, Lisabel Isaacs, Emily Chilman, Mary Cassel, Helen Sparks, Eliza Price, Emma Armstrong, Martha Reynolds, Alice Scott, Belle Gregory, Margaret Johnston, Mary A. Attwood, Clara Green, and Margaret Wardlaw. Class of 1891: Lilla Sheppard, Alice Lawson, Rachel Hanna, Kate Johnston, Agnes Kay, Lelia Batty, Emily Chilman, Bessie Dewar, Lisabel Isaacs, Mary Cassel, Helen Sparks, Martha Reynolds, Alice J. Scott, Belle Gregory, Margaret Wardlaw, Clara Green, Eliza Price, Margaret Johnson, Mary A. V. Attwood, and Emma Armstrong.

### "HONEST DOUBT."

ONE of the saddest experiences in a long illness is the doubts which creep into our minds of the goodness and mercy of God. With health and happiness for our lot, we were content to believe the truth of the Bible and what it taught, but amidst continued suffering, agony, and exhaustion, when weary days are followed by sleepless nights, the thoughts occur to our minds why am I thus? Why does God afflict me more than other people? Can He be a God of mercy and love if He keep me day after day, and week after week, in torture which appears unending? The burthen of doubt closes round us, we seem forsaken, and our faith, which at any time was never very strong, fails in the trial.

Ah! dear friends, you and I are not the only ones who have had these misgivings. Many saints have fallen into "this slough of despond," but have come out safely by the grace of God.

Job, the most patient of men, was in the same plight. In his numerous pains and losses he sought after God, but could not find Him; still, in his greatest extremity, he never let his faith slip, for he exclaims, "When He hath tried me, I shall come forth as gold" from the furnace. The doubting Thomas could not take the comfort and joy which filled the hearts of the other disciples at our Lord's Resurrection, but his heavenly Master condescended to his feeble faith, and showed him His wounded hands and side. His soul was saved, but he lost the blessing reserved for those who have not seen and yet believe.

It is faith and hope which we require in the hour of darkness. Are there not times when the sun is veiled from us for days together, and the dark fog which hangs between it and us makes us depressed and miserable? Do we despair? Do we not feel sure there is still a sun somewhere, which we hope to see again; and cannot we bear the darkness because we hope that, though it does not shine now, it will some day? It is thus with our souls. Our bodily sufferings and shattered nerves make life barely endurable, but can we not believe that our Sun of righteousness is near, and though clouds and darkness are round about Him, yet righteousness and truth are the habitation of His throne? If we can add, "My soul, hope thou in God, for I shall yet praise Him who is the health of my countenance and my God," we shall do well. But the mistake we make is to look for consolation in ourselves, in contemplating our own hearts instead of gazing upon God, and it is impossible to get comfort from what we find within us, we are so changeable. Then, again, our ideas are so limited, we are unable to see things all round or take them in their proper proportions; our individual suffering, for instance, may help to work out the universal good only we cannot grasp it. We all find it much easier to walk by sight than by faith, but our Lord's answer to all doubting enquiring souls is, "Only believe, all things are possible to him that believeth."

### The Hamilton Association for Providing Trained Male Nurses.

We have before us the rules of this association and the sixth annual report, and we have also received the satisfactory announcement that within the last month branches have been established at Hastings, West Worthing, Dartmouth, Braintree, Bayswater, Exeter, and Tunbridge Wells. As regards the regulations of the society, they are certainly very elaborate and, in many respects, admirable, but we think the charge of a guinea fee per day, for the daily nurse, is simply exorbitant. Then the demand for the first week's pay to "be made in advance" is as unnecessary as it is unique in the annals of modern nurse supply associations. Again, in rule 16 we find "The Committee reserve to themselves the right to withdraw or change a nurse at any time, on giving two clear days' notice to the employer," and this is most assuredly a piece of tyranny to which few intelligent persons would consent, nor is such an arrangement in existence, so far as we know, in any institution which sends out female nurses to private patients. Nothing but urgent family affairs, or the personal request of doctor or patient could ever justify the removal of a satisfactory attendant from a sick man, unless, of course, in a very long or trying case of illness, when a change might be mutually beneficial.

We sincerely trust that these points will receive due consideration before the issue of the next annual report, as a scheme which aims at securing the nursing of sick men by skilled and trained men, is one deserving of more general attention than has yet been accorded to it. There are many "surgical cases" which could be much more fittingly tended by a person of the same sex, and, besides these, there are many occasions and circumstances which render the services of a male attendant preferable to those of a woman. Until this fact is universally acknowledged, we suppose there will be but little progress made towards providing a proper and complete training for men.

A gentleman's valet, if experienced in providing for the personal comfort of a delicate master, has learnt for himself the first elements of private nursing; but on to these it is necessary to graft such theoretical and practical knowledge as can only be acquired in a well-managed hospital ward. But here, of course, a serious obstacle exists in the impossibility for male and female probationers to be trained together, and the only solution for the difficulty lies in the hands of the governing bodies. If the managers of one of our hospitals, or of a well-worked infirmary, could be persuaded to devote one male ward to the exclusive training of men probationers, an excellent result would follow, and we might hope, in time, to have a supply of intelligent and well-taught attendants. We know that a great many doctors strongly object to employing male nurses, preferring to engage women in all cases, and this is but natural when we compare the ordinary man with the highly-trained female nurse, whose position has become such a clearly-defined standpoint in the last half-dozen years. Properly-trained men of temperate habits and good moral tone will soon find their value acknowledged, and we already find that employers supplied from the Hamilton Association are not only willing but eager to apply to it again when the necessity for a nurse arises.

Then we must also look at the subject from the woman's standpoint. We venture to say that she will gladly welcome the day when, a sufficient number of trained men being available, she will be released from many unpleasant and trying cases, for which, at the present time, her services are frequently demanded, but if men aspire to the popularity of their sisters in the profession they must content themselves with as reasonable a rate of pay. While a perfectly-trained nurse of long experience is considered well remunerated by two guineas per week, the male nurse's terms are "from one and a-half to four guineas a-week," and we fail to see what special talent or knowledge justifies the demand for the higher sum.

### The Nurses and the Basuto War Medal.

The following letter has appeared in the *Diamond Field Advertiser*:—

Sir,—I notice in a recent issue of your paper a letter from a "Staff Officer." We are thankful for any ideas in furtherance of the war medal movement, and "Staff Officer" will be glad to hear that this question has already come before the Committee, and it is our intention to respectfully urge the Government to grant medals to those nurses who took part in the war.

I know the whole of the Committee agree with "Staff Officer's" letter, and thank him for bringing this subject to the notice of the public.

The small beginning in Kimberley is spreading beyond our most sanguine expectations. The Secretary has received letters of support from members of Parliament in London and other parts of the world, and from the Transvaal, Bechuanaland, the Free State, and Cape Colony. The Committee are so satisfied with the results up to the present that we are hoping the medal will be struck in time for the Exhibition, and that one of the functions everyone would hope to attend will, we expect, be a full dress parade of the Diamond Fields Horse, Victoria Rifles, Kimberley Scots, Cape Police, and last, but not least, the Cadets, to see the decoration conferred on our nurses by our popular Governor, His Excellency, Sir Henry Loch, K.C.M.G.—I am, &c.,

J. Salonika.

### Presentation.

Salford Royal Hospital.—Nurse Margaret Connor, of this hospital, has been presented by her fellow nurses with a beautiful bag and purse and many good wishes for her success. Nurse Connor leaves on July 15th for Sydney and is sent out by Lady Samuel.

### Notes and Queries.

To Correspondents.—1. Questions or answers may be written on post-cards. 2. Advertisements in disguise are inadmissible. 3. In answering a query please quote the number. 4. A private answer can only be sent in urgent cases, and then a stamped addressed envelope must be enclosed. 5. Every communication must be accompanied by the writer's full name and address, not necessarily for publication. 6. Correspondents are requested to help their fellow nurses by answering such queries as they can.

**Answers.**

*Sairey Gamp.*—Sisters at St. Mary's wear a navy blue dress, nurses grey beige, and probationers a small checked blue and white cotton.

*F. P.*—The Midwives' Institute is at 12, Buckingham Street, Strand; if you write, or, better still, go and see Mrs. Nicoll, the Secretary; she will give you every information.

### Wants and Workers.

[Under this heading, we propose to try whether we can be useful to our readers in making the wants of some known to others who are willing to do what work they can to aid the great cause of curing and cheering the sick. Wants can only be inserted from those who are connected with some institution or association, or who are willing to have their full name and address printed.]

District Nurse in large and scattered country parish would be very grateful for an occasional parcel of illustrated papers (any date), magazines, &c. Also old linen or calico.—*M. Mattocks, Church House, Bangor Is-y-coed, near Wrexham, North Wales.*

Has anybody a second-hand water-bed to sell or to give for a poor chronic and intense sufferer?—*Miss Fortescue, Hainton Vicarage, Lincoln.*

*Matron, Local Board Hospital, Allen Croft, Bolsterstone, near Sheffield*, will be glad if anybody will send her any books or papers for the use of convalescents; at present they have none of any sort.

### Small-Pox and Infectious Fever Nursing.

Some correspondence in our columns under the heading of the "Needless Dread of Nursing Small-Pox Cases," illustrates the existence of an old prejudice, which was due mainly to insanitary surroundings. We have known a local sanitary authority to be so panic-stricken by the sudden appearance of a case of small-pox, that they engaged a large hearse into which the patient was put and then drawn into an open field. Strange to say, the case made a perfectly good recovery. We have had a large personal experience of small-pox, and have lived in the midst of it for many weeks. The result of our experience goes to prove that a small-pox ward can be made perfectly wholesome and pleasant, and that it need differ but little, if at all, from an ordinary ward, so far as the staff is concerned. If nurses are first re-vaccinated, if they are allowed regular periods for exercise and fresh air, and providing they have adequate sleeping accommodation and possess common sense, which last is most important of all, there is practically no risk in nursing small-pox. The best disinfectant and the best preventive to infection is an abundance of fresh air. A ward may be disinfected after a case of small-pox in any way known to science, every article may be destroyed, the whole of the walls and woodwork may be repainted and purified, the chimneys be swept, and yet small-pox will recur so soon as patients are again admitted. On the other hand, if a ward after a case of small-pox, be left unoccupied for three or four weeks, during which fresh air is freely passed through it, and if it be then disinfected, no renewal of the outbreak need be feared. In the same way barrack wards of the simplest kind of one storey each, which contain at least 2,000 cubic feet per bed, and have gas jet ventilators in the roof throughout its whole extent, with plenty of free inlets at the sides and ends of the ward, will always be found sweet even when the beds are full of confluent cases. Of course, any sanitary authority which fails to provide adequate accommodation for small-pox patients should be compelled by law to nurse those cases themselves; and if they contracted the disease, so much the better. No nurse should consent to undertake the charge of small-pox cases unless the accommodation is adequate, as it will usually be found to be in this country at the present time. It would be a waste of sympathy to condole with the inhabitants of any district not possessing suitable hospital accommodation for small-pox cases, because the law enables them to secure it without difficulty by the exercise of the minimum of public spirit on their part. To sum up "Veritas" is right in declaring that the danger of nursing small-pox under proper conditions is practically nil, and that dread referred to by "J. M." arises from prejudice or ignorance due to a want of training in small-pox nursing for which the teachers must be held responsible. Indeed, J. M. admits that experience not only dispels all fear, but excites the wish for a greater opportunity of attending these cases. It may be added, in large towns where the friends were formerly allowed to undertake the nursing of confluent cases at their own homes, many patients used to die of sheer starvation due to a distaste of food on the part of the patient and the absence of a trained nurse to secure its regular administration. Hence confluent cases are the very ones where a trained nurse can be of more real service than all the doctors and physic in the world. For this reason nurses, we are sure, will agree that, should the opportunity offer, they will cheerfully undertake to nurse confluent cases of small-pox without fear or dread, and with a devotion which will save many lives.

We have had several complaints lately of the character of the nursing at the fever hospitals under the management of the Metropolitan Asylums Board. It is stated that the patients are not properly washed or attended to, and that the children, when convalescent, are sent to their homes in a dirty condition. We shall content ourselves at the moment with calling attention to the statement, and requesting the Local Government Board to send an inspector to each of these establishments to ascertain the facts. Should such neglect be found to exist, then the whole system of engaging and employing nurses under the Metropolitan Asylums Board, which is admittedly imperfect, will have to be promptly re-organised.

### Ring-Worm and Mob Caps.

Of the many "ills which flesh is heir to," ring-worm is by no means the least troublesome, for it is very slow and obstinate in yielding to treatment, besides being extremely infectious. The child of gentle birth by contracting it, at once becomes an alien, isolated from all young companions until he might almost fancy himself in some way to blame for his misfortune. This kind of case, happily, is not frequent, but the same unpleasant disease is well known in poor or unclean homes, and in certain classes of schools where the mischief is soon widely spread. When a child is discovered to be thus afflicted in a workhouse school, he is promptly transferred to the infirmary, and, to tell the truth, these little waifs and strays of humanity rather like the change thus secured to them. At that most admirable infirmary (St. Pancras) on Highgate Hill, we saw two little girls in a women's ward who were under treatment, and the Sister had constructed the prettiest of little white caps, each child having a distinctive rosette in front. The general effect was distinctly becoming, showing that a bit of lint without, and tissue paper within, can, in tasteful fingers, form as efficacious a head-covering as yards of unsightly bandaging.

### The Metropolitan Provident Medical Association.

A meeting of the Metropolitan Provident Medical Association held June 23rd, at Lady Stanley of Alderley's house in Dover Street, was well attended. Sir Spencer Wells in the chair, proved himself a warm advocate and explained the scheme at some length. Letters expressing sympathy with the movement and regretting unavoidable absence were read from Lord Derby, Sir Henry Ackland, Hon. Maud Stanley, Mr. Barnet, Miss Twining, Lord Sandhurst, Rev. Erskine Clarke, and many others. Mr. Buxton spoke of the satisfactory progress made by the Provident Dispensary at Whitechapel, and the friendly relations maintained between it and the London Hospital. Sir Charles Freemantle, Miss F. Davenport Hill, Mr. Claud Montefiore, Mr. Mocatta, Mrs. Dacre Craven, and other speakers were listened to with attention, and Mrs. Garrett Anderson was not only interesting, but amusing, as she insisted on giving prominence to both sides of the question. Taken together the addresses certainly proved conclusively that there remains ample scope for the Provident Medical Association to continue in its usefulness which aids, and certainly in no way hinders, the valuable work done in hospital "in" and "out-patient" departments. We were glad to read in the report that the question of proper nursing for the members of the Association is receiving special attention. The proceedings on Thursday closed with a vote of thanks to Lady Stanley for her courteous hospitality, which she personally acknowledged in a few pleasant words.

### Four Months in a Hospital Ward.

### A PERSONAL EXPERIENCE IN A PROVINCIAL HOSPITAL—VII.

### "Darker Shadows."

And now something of the "darker shadows" of this hospital life, where all is necessarily sombre when viewed in comparison with the brightness and light of outside health and strength. It was ten o'clock on the second night after my arrival that I noticed the entrance of two respectably dressed women and a man, who were ushered to the bedside of the poor consumptive patient, whose sufferings had impressed me so deeply on my first coming into the ward. The charge nurse brought them in, and I knew instinctively that they had come to take a "last farewell" of their sinking relative. Screens were placed around his bed, and so some measure of privacy was given to the grievous sufferer and his friends in that agonizing hour, but amid the silence of the now sleeping ward, his laboured breathing and the sobs of the watchers were plainly audible. The dim lights and the flickering of the fire heightened beyond description the awful character of the scene, and I felt as I have never felt before in my life. Presently the sound of the hard drawn breathing grew fainter and fainter, until it ceased altogether, and the departure of the weeping mourners told me that one of us had gone down into "the valley of the shadow of death."

### "Johnnie."

One day a little boy about ten years of age, named Johnnie, was brought in, very, very ill of some lung disease; he was wasted to a skeleton, and his face, as white as the driven snow, was almost transparent. The doctors had some hopes of him, and every nerve was strained by the nurses to pull him through, but it was not to be. His mother came often to see him, and the little fellow used to cheer up and smile when he saw her coming. At last, after about three weeks, he was evidently going fast, and he seemed conscious of it, for when tender-hearted old Darky was raising him up on his pillow one night to make him easier, the child said: "Darky, do you think I shall live till my mother comes to-morrow?" Darky's eyes filled with tears, it was the only answer he could make, but the dying boy understood it, for he said, "Then I shall see her in again heaven," and so may it be! for when next morning the mother, whose home was some distance away and who had to work hard for six other fatherless children came to see her sick darling, his spirit had flown.

### "Thoughtful Kindness."

A list is kept at the porter's lodge of the names of those patients whose lives are considered by the doctors to be in danger, in order that the relatives of such patients may be admitted at any time, irrespective of "visitors' days," a most humane arrangement, and one that proves clearly how excellent is the spirit that runs through the management of this institution. In all the cases that ended fatally in that ward while I was there, every effort was made by telegram and by special messenger to bring the friends of the departing one to his bedside.

### I Change my Place of Rest.

It was now ten weeks since I had first laid me down on my narrow bed, and one day, owing to my having grown very weak and unable to sleep, the house doctor ordered my removal to a smaller ward containing a single bed, in the hope that the extra quiet would give me a better chance of recovery. It was an act of kindness on his part which affected me deeply, and I shall ever think of that quiet, unassuming doctor with affection, for hope was waning fast, and I had begun to fear that my turn for the dread screens was at hand. They brought an armchair on wheels to my bedside, and, wrapped in blankets, they helped me into it, and wheeled me slowly down the ward, and I looked my "Good-bye" to my comrades as I passed along. Whether or not they were sorry to lose me I cannot tell, but that I was sorry to lose them I do know from the tell-tale choking sensation that came over me; but it was soon gone, and I went out down the corridor, a probationer following with my "locker," to the small ward, where I was quickly ensconced in a similar bed to that I had left.

### "I have Visitors."

That night I slept better than I had done for a fortnight, and the following days saw the flickering flame reviving. I soon found that I was not by any means to be left "alone," or, at least, if a "hermit," I was a hermit whose "cave" was to be frequently visited. An hour rarely elapsed without one or other of the nurses coming to exchange a few words with me, and sometimes a convalescent from the big ward would come in and have a chat or bring a draughtboard for my amusement, but this latter recreation I found too wearying, and so they just used to sit down at my bedside and tell me some of their experiences.

### "A Ray of Hope."

Very slowly, from day to day, I felt my strength returning, and in fear and trembling I would scan the doctors' faces for confirmation of what I felt, as they came their punctual rounds, until at last I could detect an unmistakeable look of satisfaction in their faces, which, in a few days, broadened into a downright smile when they gave me the welcome permission to "sit up." Anyone who has been long ill, and has gone through the ordeal of being told professionally that there is "no hope," will know something of the joy and gladness with which I received this evidence of returning life, for long before I entered this hospital I had received the "sentence of death" at the hands of a doctor.

Feeble and tottering at first, I now gained ground apace, and in a week I was able to revisit the scene of my first hospital experience, the big ward, and claim my share of those voluntary duties so eagerly sought for by all the convalescents. Open air exercise was the next comforting order I received, and my old friend Darky considered it his special duty to lend me the support of his arm on this my first appearance among the fortunate ones, who, like tender plants long under artificial conditions, were put out for a few hours each day, to "harden" as it were, before being finally restored to the outside world.

### My "Surgical" Friends.

Here I met men from other wards, and had yet fresh opportunities of reading character. The surgical men were very "surgical," and it required an effort to get them away from the subject of knives, chloroform, lancets, and bandages; but once started on their own particular calling in life, a rich fund of useful and interesting information would follow. Sometimes our exercising ground was enlivened by the presence of two or three convalescent children, picturesquely clad in red hoods and capes, and it was a goodly sight to watch the house doctors playing with these little ones and giving them rides round and round the lawn on tricycles. Pleasant would be the recollections of those children of at least this stage of their illness, and kindly must have been the hearts of those doctors who thus devoted a half-hour of their hard earned recreation to the amusement of the youthful exiles for whom they had already done so much.

*(To be continued.)*

## Figures and Tables

**Figure f1:**
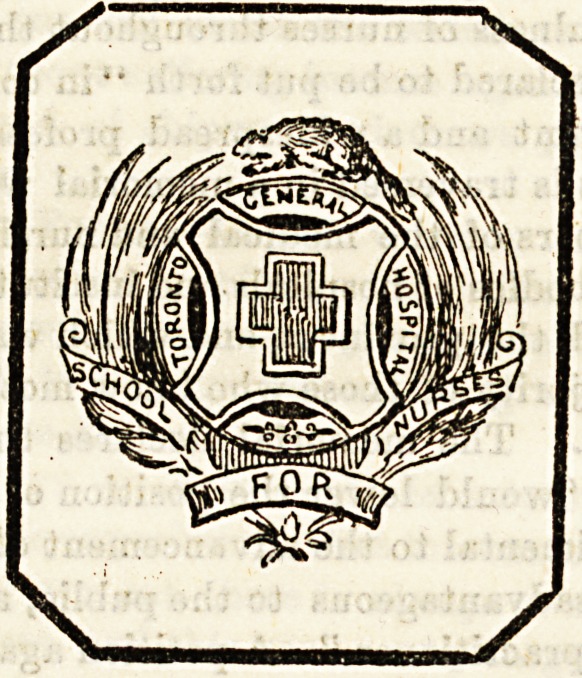


**Figure f2:**